# The effectiveness of Stepping stones Triple P: the design of a randomised controlled trial on a parenting programme regarding children with mild intellectual disability and psychosocial problems versus care as usual

**DOI:** 10.1186/1471-2458-11-676

**Published:** 2011-08-30

**Authors:** Marijke Kleefman, Daniëlle EMC Jansen, Sijmen A Reijneveld

**Affiliations:** 1 Department of Health Sciences, University Medical Center Groningen, University of Groningen, PO Box 196, 9700 AD Groningen, the Netherlands; 2 Department of Sociology and Interuniversity Center for Social Science Theory and Methodology (ICS), University of Groningen, the Netherlands

## Abstract

**Background:**

Children with an intellectual disability are at increased risk of psychosocial problems. This leads to serious restrictions in the daily functioning of the children and to parental stress. Stepping Stones Triple P aims to prevent severe behavioural, emotional and developmental problems in children with a (intellectual) disability by enhancing parenting knowledge and skills, and the self-confidence of parents. This paper aims to describe the design of a study of the effectiveness of parenting counselling using Stepping Stones Triple P compared to Care as Usual.

**Methods/Design:**

The effects of Stepping Stones Triple P will be studied in a Randomised Controlled Trial. Parents of children aged 5-12 years with an IQ of 50-85 will be recruited from schools. Prior to randomisation, parents complete a screening questionnaire about their child's psychosocial problems and their parenting skills. Subsequently, parents of children with increased levels of psychosocial problems (score on Strengths and Difficulties Questionnaire ≥ 14) will be invited to participate in the intervention study. After obtaining consent, parents will be randomised either to the experimental group (Stepping Stones Triple P) or to Care as Usual. The primary outcome is a change in the child's psychosocial problems according to parents and teachers. The secondary outcome is a change in parenting skills. Data will be collected before the start of the intervention, immediately after the intervention, and six months after.

**Discussion:**

This paper presents an outline of the background and design of a randomised controlled trial to investigate the effectiveness of Stepping Stones Triple P, which aims to decrease psychosocial problems in children with a mild intellectual disability. Stepping Stones Triple P seems promising, but evidence on its effectiveness for this population is still lacking. This study provides evidence about the effects of this intervention in a community-based population of children with a mild intellectual disability.

**Trial registration**

Netherlands Trial Register (NTR): NTR2624

## Background

Psychosocial problems, such as problems with behaviour, emotions and relationships, occur frequently in children with an intellectual disability (ID). Estimates of their prevalence rates vary widely, from 30% to over 60% [1-3]. The combination of psychosocial problems and ID significantly limits occupational opportunities in the post-school period and can also lead to major restrictions in participation in educational and recreational programmes [4]. Moreover, a child's psychosocial problems and parenting stress exacerbate each other over time [5]. Further, the presence of psychosocial problems also leads to parental stress due to parenting challenges [6,7].

The improvement of parenting skills by parenting interventions may lead to a significant reduction in both the psychosocial problems of the child and the parental stress [8]. A promising parenting programme that may help to reduce these problems in these children is Stepping Stones Triple P (SSTP). SSTP aims to prevent severe behavioural, emotional and developmental problems in children with a disability by enhancing the knowledge, skills and confidence of parents [9]. However, although SSTP looks promising, there is little strong evidence on its effectiveness. There are two studies in Australia in preschool-aged children with developmental disabilities and problem behaviour [10,11]. Furthermore, there is a study in Australia on children diagnosed with autism spectrum disorders [12]. The results of these studies demonstrate maintained significant improvements in child behaviour and parenting styles, and parents reported a high level of satisfaction with SSTP. A recent Dutch study on SSTP without control groups shows positive effects on the child's psychosocial problems, on parenting skills, family functioning and parental wellbeing [13]. However, there is no evidence with Randomised Controlled Trials (RCT) for SSTP among this broad target population of children with mild ID and their parents.

The aim of this study is to assess the effectiveness of SSTP regarding the reduction of a child's psychosocial problems and parental stress among children with mild ID and their parents. This paper describes the design of the evaluation of the effectiveness of parenting counselling according to SSTP regarding the psychosocial problems of children with mild ID compared to a control group receiving Care as Usual (CAU).

## Methods/Design

### Trial design

**Figure 1:**
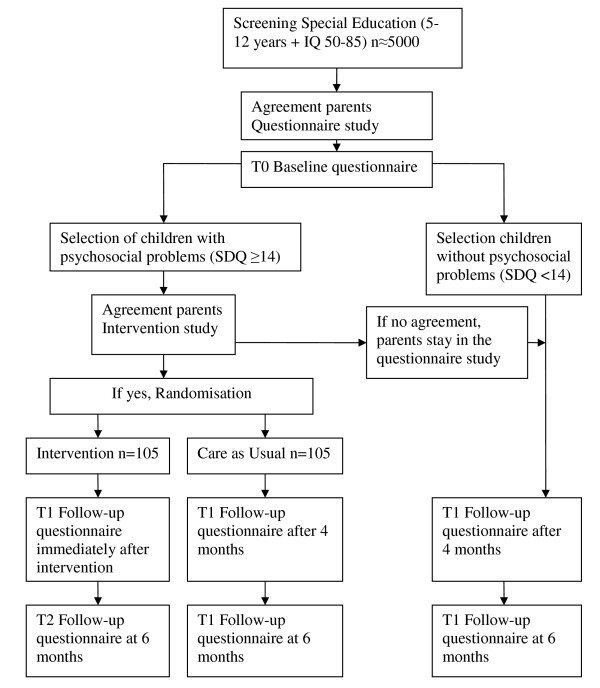
**Study design**. This figure describes the design of an RCT on the effectiveness of parenting counselling according to SSTP compared to a control group receiving CAU.

The design of the study will be described following the CONSORT guidelines [14]. The study will be conducted as an RCT on the effectiveness of parenting counselling according to SSTP compared to a control group receiving CAU (Figure 1). There will be a screening before the intervention (T0), an assessment immediately after the intervention (T1) and a follow-up assessment after six months (T2). Parents will participate voluntarily in this study and are free to leave the study at any time. All parents will provide written informed consent before they participate in the study. Ethical permission for this study has been obtained from the Medical Ethics Committee of the University Medical Center Groningen. The study will be performed between October 2010 and December 2013.

### Participants

Via schools, parents of children aged 5-12 years with an IQ of 50-85, living in the four Northern provinces of the Netherlands (Groningen, Friesland, Drenthe and a part of Overijssel) will be invited to fill out a screening questionnaire about their child's psychosocial problems and their parenting skills. The majority of children with mild ID attend three types of school for special educational needs in the Netherlands, in Dutch called 'SBO', 'REC3' and 'REC4'. SBO ('Speciaal Basis Onderwijs'; special primary education) comprises children with mild intellectual disabilities (IQ of between 70 and 85), learning difficulties and/or behaviour difficulties. REC 3 ('Regional Expertise Center cluster 3') includes children with physical disabilities, (mild) ID (IQ < 55 or IQ between 56 and 70 with other severe disabilities), and/or chronic diseases. REC 4 ('REC cluster 4') includes children with psychiatric and/or behavioural disorders with or without mild ID (IQ > 70) [15,16]. Participating schools expect that 30% of the REC4 pupils, 30% of the REC3 pupils and 95% of the SBO pupils will be eligible for screening (age 5-12 years and IQ 50-85).

In order to increase response rates, we will offer assistance if required to fill out the screening questionnaire and will visit all families to fill out the second and third questionnaires [1]. Exclusion criteria are: (1) the child lives in residential care (except foster care), (2) parents are unable to speak Dutch, (3) information about the child's IQ is not available, and (4) parents live outside the research area.

When parents report psychosocial problems in their child at T0, they will be invited to participate in the intervention study. Exclusion criteria are: (1) a brother or sister (with a higher Strengths and Difficulties Questionnaire (SDQ) score) is participating in the study, and (2) the parents are receiving treatment for parenting skills or other treatment that potentially clashes with SSTP.

### Intervention

SSTP is a family intervention and aims to prevent severe behavioural, emotional and developmental problems in children with all kinds of disabilities, including mild ID, by enhancing the knowledge, skills and confidence of parents [9]. This method is part of the Australian Triple P - Positive Parenting Program, a system of parenting and family interventions for parents of children who have or are at risk of psychosocial problems [17]. There are seven principles of parenting in SSTP: (1) ensure a safe interesting environment, (2) create a positive learning environment, (3) use assertive discipline, (4) have realistic expectations, (5) take care of oneself as a parent, (6) family adaptation to having a child with a disability, and (7) be part of the community. The last two principles are additional ones, specifically related to the parenting of children with a disability [10].

SSTP takes 8-10 individual sessions, provided over 10-12 weeks. Each session typically lasts 60-120 minutes. The first module, 'Assessment', consists of two sessions of about 60-90 minutes. In this module, parents formulate hypotheses about the problems and make relevant causes and factors clear. The second module, 'Positive Parenting', also consists of two sessions of about 60-90 minutes. In these sessions parenting strategies should be introduced to parents. The third module, 'Practice', consists of three sessions of about 40-60 minutes. In these sessions, parents will practise the parenting strategies and receive support. The final module, 'Planned Activities Training', consists of three sessions. Parents will be assisted in the practical implementation of the strategies [9].

### Control condition

Participants assigned to the control condition may utilise any service, except SSTP. These services can be anything aimed to alleviate parental stress or the psychosocial problems of the child. CAU can consist of Practical Pedagogical Family Support (PPG), Video-home training (VHT), Intensive Pedagogical Homecare (IPT) or Intensive Orthopedagogical Family Care (IOG), but also of psychiatric or psychological care for the child and, in some cases, no care at all [18].

### Outcome measures

The primary outcome is the child's psychosocial problems measured by the SDQ (parent and teacher versions) and the Eyberg Child Behaviour Inventory (ECBI). The SDQ is a 25-item questionnaire, covering emotional symptoms, conduct problems, hyperactivity, peer relationship problems, pro-social behaviour and the impact of the problems on functioning [19]. We will use two informants to measure each child's psychosocial problems (i.e. the teacher and the parent). Multiple informants on a child's psychosocial problems are invaluable for understanding the home and community functioning of children with mild ID, because psychosocial problems may be highly situational and differ at school and at home [20-22]. The ECBI, a 36-item questionnaire, will be used to measure parental perceptions of the disruptive behaviour of the child, including frequency and its identification as a problem [23].

The secondary outcome is parenting skills measured by the Parenting Stress Index (PSI) and the Alabama Parenting Questionnaire (APQ). The PSI measures parents' perceived stress due to parenting. The short 25-item version of the PSI comprises questions on experience of parenting leading to a total 'parenting stress' score [24]. The APQ measures parenting practices. The short version of the APQ consists of 35 items categorised into four subscales: parental involvement, positive parenting, poor monitoring and inconsistent discipline [25].

### Other variables

In addition, data will be collected about the current use of health care and use of health care in the past (in the preceding four weeks, six months and longer than six months ago). Moreover, questions will be asked regarding the need for parental support and satisfaction with care.

Data on sociodemographic characteristics (parental education, family income, family structure, ethnicity, parental age and parental employment) and child medical conditions will be collected at baseline to adjust for potential differences between the intervention and control groups.

### Sample size

The parental SDQ score will serve as the primary outcome measure to determine the sample size. For a 3-point decrease in the SDQ total score, given a standard deviation (SD) of the SDQ of 6 points (an effect size of 0.5), at alpha = 0.05 (two-sided) and beta = 0.20, 63 children need to be included in each group (SSTP and CAU). With adjustment for a 'loss to follow-up' of about 40%, 210 children need to be included in the study, 105 children in each treatment condition.

Parents will be included if their child has an SDQ total problems score of 14 or higher. The prevalence rate of an SDQ ≥ 14 among children who are not under current treatment for their mental health problems is estimated at 55% [2]. Therefore, (2 × 105)/55% = 381 children with mild ID aged 5-12 years are required. Adjusting for 30% refusal to participate and 10% incomplete SDQs, 635 parents will have to fill in the SDQ.

### Randomisation procedure

After informed consent by the parents and application of the exclusion criteria by the researcher, the parents will be randomised to either SSTP or CAU. The randomisation will be based on a computer-generated randomisation algorithm. Individuals will be randomised per centre in each of the four participating centres in mixed blocks of four and six to prevent unequal randomisation within the centres [26]. If parents are randomised to SSTP, the intervention will start within four weeks of administering the screening questionnaire. Blinding will be imposed on the teachers, i.e. they will not know who is participating in which group. The SSTP trainers cannot be blinded to treatment status during the intervention, because blinding is apparently impossible.

### Statistical analyses

The results will be analysed on an intention to treat principle with all randomised participants, using all follow-up measures. Consequently, participants who filled out the questionnaire will be included in the analyses, regardless of whether or not they have completed the intervention.

The baseline characteristics of the parents in both research groups will be compared using Chi-squared tests for nominal and ordinal variables, and t-tests for continuous variables. The differences between the intervention and control condition over time will be assessed by mixed model techniques to consider factors that may influence the outcomes. If necessary, data on subjects lost to follow-up will be handled by extrapolation of the last observation or by sophisticated imputation techniques. Changes in the child's psychosocial problems and parenting characteristics will be expressed as standardised effect sizes with 95% confidence intervals. The CONSORT guidelines will be followed [14]. All the analyses will be carried out using SPSS 17.0.

## Discussion

This paper presents an outline of the background and design of an Randomised Controlled Trial (RCT) to assess the effectiveness of Stepping Stones Triple P (SSTP) in children with a mild intellectual disability (ID) and psychosocial problems.

The majority of children with mild ID also have psychosocial problems. In the long term, these problems can increase and cause distress, disturb family life and interfere with other everyday activities [27]. The combination of psychosocial problems and mild ID can be a major cause of reduced participation and social integration. This can lead to problems in the future, for example problems in living in society and being autonomous [4]. SSTP is a parenting support programme that aims to decrease psychosocial problems in children with mild ID. However, as yet there does not seem to have been an RCT for SSTP.

This study will contribute to science in that it will provide empirical evidence on the effect of SSTP on psychosocial problems at home and at school in children with mild ID. Decreasing a child's psychosocial problems will lead to better development and a strengthening of the child's participation [28].

### Strengths

The study will be conducted in an experimental design. Therefore, the risk of selection and allocation bias will be minimised and the internal validity will be high. Random allocation ensures no systematic differences between the SSTP or Care as Usual (CAU) groups in factors, known and unknown, that may affect the outcome.

The SSTP intervention will be carried out by a Dutch healthcare organisation especially for adults and/or children with a disability or chronic illness (in Dutch: MEE). The intervention will be carried out in daily practice, and thus the study will also provide evidence on the feasibility of the implementation of SSTP.

Another strength of this study is the use of two informants in order to measure the child's psychosocial problems (i.e. teacher and parent). Multiple informants on the Strengths and Difficulties Questionnaire (SDQ) are invaluable for understanding the home and community functioning of children with mild ID, because psychosocial problems may be highly situational and differ at school and at home [20-22].

Furthermore, the potential participants will be recruited by schools for special education. In this way, the majority of parents of children with mild ID will be reached and can participate in the study [15]. In addition, due to their personal contact with parents, schools can play an important role in motivating parents to participate in the study. A representative sample will increase the external validity and conclusions will be able to be generalised. Finally, teachers will be blind as to whether a family is in the intervention or control group, which will prevent any information bias.

### Limitations

The study also has some potential limitations. Selective dropout may occur in the intervention group, i.e. a certain proportion of parents may not complete the study. Also, selective non-response may occur in the screening. People with lower incomes, more family problems and a lower IQ are at a higher risk of dropout or non-response. For example, parents with mild ID need a lot of practical support and research activities like filling out questionnaires may be difficult for them [29]. To prevent such selective non-response, we may assist parents in filling out the questionnaires.

Moreover, loss to follow-up may be high in this target population, potentially leading to biased results. To prevent this bias, all parents will receive assistance in filling out the questionnaire if they participate in the intervention study.

Another potential limitation is that we will not observe the parents' interaction with their child. Direct observation would provide the opportunity to precisely register the activities and behaviour of parents and their children during the intervention period. However, this method is costly and labour-intensive for both researchers and families [30]. Moreover, the observations themselves may affect the parent-child interactions, and may thus be a somewhat biased alternative as well.

Finally, in the study there is a short follow-up period of six months because studies about the effectiveness of parenting programmes establish findings at least on the short-term outcomes of family functioning, parenting skills and a child's psychosocial behaviour [31]. Nevertheless, this intervention aims to prevent developmental problems in children in the future, which leads to a limitation of this study being the short follow-up period. To determine long-term effects, there should be follow-up after a couple of years to assess the effects when the child is older [32].

## Conclusions

Children with psychosocial problems and mild ID are at risk of psychosocial problems and consequently problems with participation and social integration. The SSTP programme seems a promising intervention to decrease these problems, but evidence on its effectiveness for this broad population is still lacking. This study will lead to evidence-based information about the effects of this intervention in a broad population at home and at school. Its results may support public interventions to decrease the large burden due to a child's psychosocial problems for children with mild ID and their parents.

## Competing interests

The authors declare that they have no competing interests.

## Authors' contributions

SAR and DEMCJ had the original idea for the project, wrote the study proposal and obtained funding for the study. MK and DEMCJ wrote the study protocol, which was discussed by all authors leading to the final design. MK wrote the final manuscript, which was discussed, edited and revised by all authors. All authors read and approved the final manuscript.

## Pre-publication history

The pre-publication history for this paper can be accessed here:

http://www.biomedcentral.com/1471-2458/11/676/prepub
